# Surgical referrals in Northern Tanzania: a prospective assessment of rates, preventability, reasons and patterns

**DOI:** 10.1186/s12913-020-05559-x

**Published:** 2020-08-08

**Authors:** Desmond T. Jumbam, Gopal Menon, Tenzing N. Lama, William Lodge II, Sarah Maongezi, Ntuli A. Kapologwe, Isabelle Citron, David Barash, John Varallo, Erin Barringer, Monica Cainer, Mpoki Ulisubisya, Shehnaz Alidina, Boniface Nguhuni

**Affiliations:** 1 Program in Global Surgery and Social Change, Department of Global Health and Social Medicine, Harvard Medical School, Boston, MA USA; 2 Department of Plastic and Oral Surgery, Boston Children’s Hospital, Boston, MA USA; 3 Ministry of Health, Community Development, Gender, Elderly and Children, Dodoma, Tanzania; 4 President’s Office, Regional Administration and Local Government, Dodoma, Tanzania; 5 GE Foundation, Boston, MA USA; 6 Jhpiego, Baltimore, MD USA; 7 Dalberg Advisors, New York, NY USA; 8 Assist International, Ripon, CA USA

**Keywords:** Health systems, Referral systems, Surgical systems, Referral rate, Reasons for referral, Referral patterns

## Abstract

**Background:**

An effective referral system is essential for a high-quality health system that provides safe surgical care while optimizing patient outcomes and ensuring efficiency. The role of referral systems in countries with under-resourced health systems is poorly understood. The aim of this study was to examine the rates, preventability, reasons and patterns of outward referrals of surgical patients across three levels of the healthcare system in Northern Tanzania.

**Methods:**

Referrals from surgical and obstetric wards were assessed at 20 health facilities in five rural regions prospectively over 3 months. Trained physician data collectors used data collection forms to capture referral details daily from hospital referral letters and through discussions with clinicians and nurses. Referrals were deemed preventable if the presenting condition was one that should be managed at the referring facility level per the national surgical, obstetric and anaesthesia plan but was referred.

**Results:**

Seven hundred forty-three total outward referrals were recorded during the study period. The referral rate was highest at regional hospitals (2.9%), followed by district hospitals (1.9%) and health centers (1.5%). About 35% of all referrals were preventable, with the highest rate from regional hospitals (70%). The most common reasons for referrals were staff-related (76%), followed by equipment (55%) and drugs or supplies (21%). Patient preference accounted for 1% of referrals. Three quarters of referrals (77%) were to the zonal hospital, followed by the regional hospitals (17%) and district hospitals (12%). The most common reason for referral to zonal (84%) and regional level (66%) hospitals was need for specialist care while the most common reason for referral to district level hospitals was non-functional imaging diagnostic equipment (28%).

**Conclusions:**

Improving the referral system in Tanzania, in order to improve quality and efficiency of patient care, will require significant investments in human resources and equipment to meet the recommended standards at each level of care. Specifically, improving access to specialists at regional referral and district hospitals is likely to reduce the number of preventable referrals to higher level hospitals, thereby reducing overcrowding at higher-level hospitals and improving the efficiency of the health system.

## Background

A functioning health system is one that delivers high-quality healthcare services to all people, when and where they need them [1]. High-quality healthcare includes “a thorough assessment, detection of symptomatic and coexisting conditions, accurate diagnosis, appropriate and timely treatment, referral when needed for hospital care or surgery and the ability to follow the patient and adjust the treatment course as need” [2]. An effective referral system is a prerequisite for high-quality healthcare and is needed to optimize patient outcomes while ensuring that available resources are used efficiently [2–4]. Effective referral systems help recognize conditions that require an escalation of care to more specialized health facilities and ensure that they are managed in a timely and comprehensive manner at the appropriate level.

Referral systems that do not function as intended due to inadequate capacity at lower levels of care, encourages referral to higher-level centers, even for uncomplicated conditions that should be managed at lower levels. As such, lower-level centers become underutilized resulting in overburdened high-level centers with inadequate resources to care for the complex conditions for which they are designed. This leads to inefficiencies at all levels within health systems with ineffective referral pathways [5, 6]. Referral to distant hospitals also negatively affects patients, leading to delays in receiving care, significant direct costs for care and indirect costs with time away from work [7–9].

Effective referral systems are crucial for surgical care delivery. About a third of the global burden of disease requires surgical expertise with low-income and middle-income countries (LMICs) affected most severely [10–12]. Surgical referrals are patient referrals for conditions that require surgical expertise for diagnosis, management and treatment. Surgical care requires efficient referral systems as it encompasses a wide range of conditions. Common and simple conditions, such as appendicitis, should be addressed at lower-level facilities. On the other hand, more complex surgical conditions, such as reconstructive surgery, require specialized surgical teams, more suited to higher-level facilities. The significant burden of surgical conditions in LMICs combined with the nascent and under-resourced surgical ecosystem makes an effective referral system indispensable. However, patient referral systems in LMICs remain ineffective due to inadequate capacity at primary and secondary care levels, self-referrals (patient bypass primary and secondary care levels to seek care at tertiary care levels) and poor coordination of the referral system [13–16].

In 2015, the Ministry of Health, Community Development, Gender, Elderly and Children (MoHCDGEC) of Tanzania launched its first National Surgical, Obstetric and Anesthesia Plan (NSOAP) to improve access to safe, timely and affordable surgical care for Tanzanians by 2025 [17]. A priority of this NSOAP is strengthening the referral system. However, there is little available data regarding the rates, preventability, reasons, and patterns of referrals needed to inform the implementation of this policy.

Few studies have been conducted to assess the patient referral system in Tanzania. In 2004, Simba and colleagues found that up to 70% of admitted patients at Muhimbili National Hospital, a tertiary hospital in Dar es Salaam, were self-referred, with the majority requiring surgical intervention [13]. In examining the effectiveness of the maternal referral system in Rufuji District, Pembe and colleagues found that compliance with referral advice was low due to financial constraints and difficulty finding transportation to the referred facility [18]. Though insightful, these studies only examine referrals from the perspective of the receiving facility as opposed to the referring centres and, as such, do not provide a holistic picture of the challenges of the referral system.

The aim of this study was to assess the capacity of referral system at three levels of the healthcare system in Northern Tanzania. Specifically, we sought to assess the *rates, preventability, reasons, and patterns* of outward referrals of patients from surgical and obstetric wards at health facilities in Northern Tanzania. Findings generated are intended to provide guidance on the implementation of the NSOAP and contribute to health systems knowledge in Tanzania.

## Methods

### Setting

The United Republic of Tanzania is a lower middle income country located in Eastern Africa with a population of 56 million and a gross domestic product per capita of 1,122 USD [19]. Life expectancy at birth is 65 years and the maternal mortality is 556 per 100,000 live births [20]. The country is administratively divided into seven zones, which are further sub-divided into 26 total regions.

The Lake Zone surrounds the southern shore of Lake Victoria and borders Kenya, Uganda, Rwanda and Burundi. It is divided into six regions; Mwanza, Kagera, Mara, Shinyanga, Geita and Simiyu. All regions in the Lake Zone, except Mwanza Region, were included in this study. The population of these 5 regions is approximately 9 million people. Bugando Medical Center, the zonal hospital, is located in Mwanza region and serves a catchment population of over 14 million [21]. Cumulatively, the study regions have 1196 health facilities; 1021 dispensaries, 137 health centers, 18 district hospitals, 5 regional referral hospital and 15 other hospitals at the time of the study [22].

**Fig. 1:**
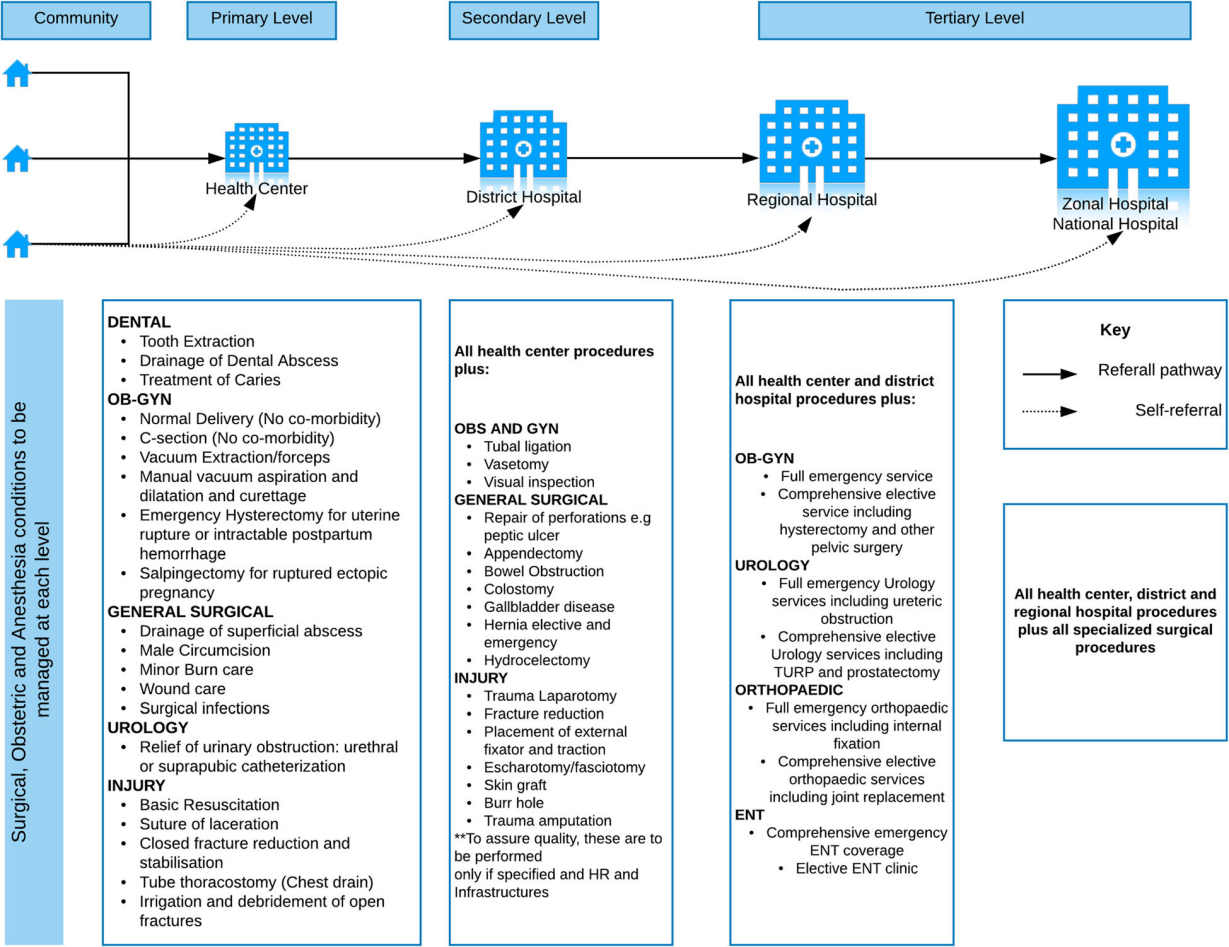
Referral Pathway of the Tanzania healthcare delivery system. This figure was created by the authors using Keynote version 9.0.1 (6196)

The healthcare system in Tanzania is structured such that health services begin at the community level and patients are referred up the referral chain based on the complexity of services needed. Figure 1 illustrates the referral pathway along with surgical procedures to be provided at each level of care according to the NSOAP.

In order for this system to function efficiently, patients must move up and down the referral pathway based on the complexity of care needed with few patients bypassing lower level facilities to higher level facilities.

### Sample

**Fig. 2:**
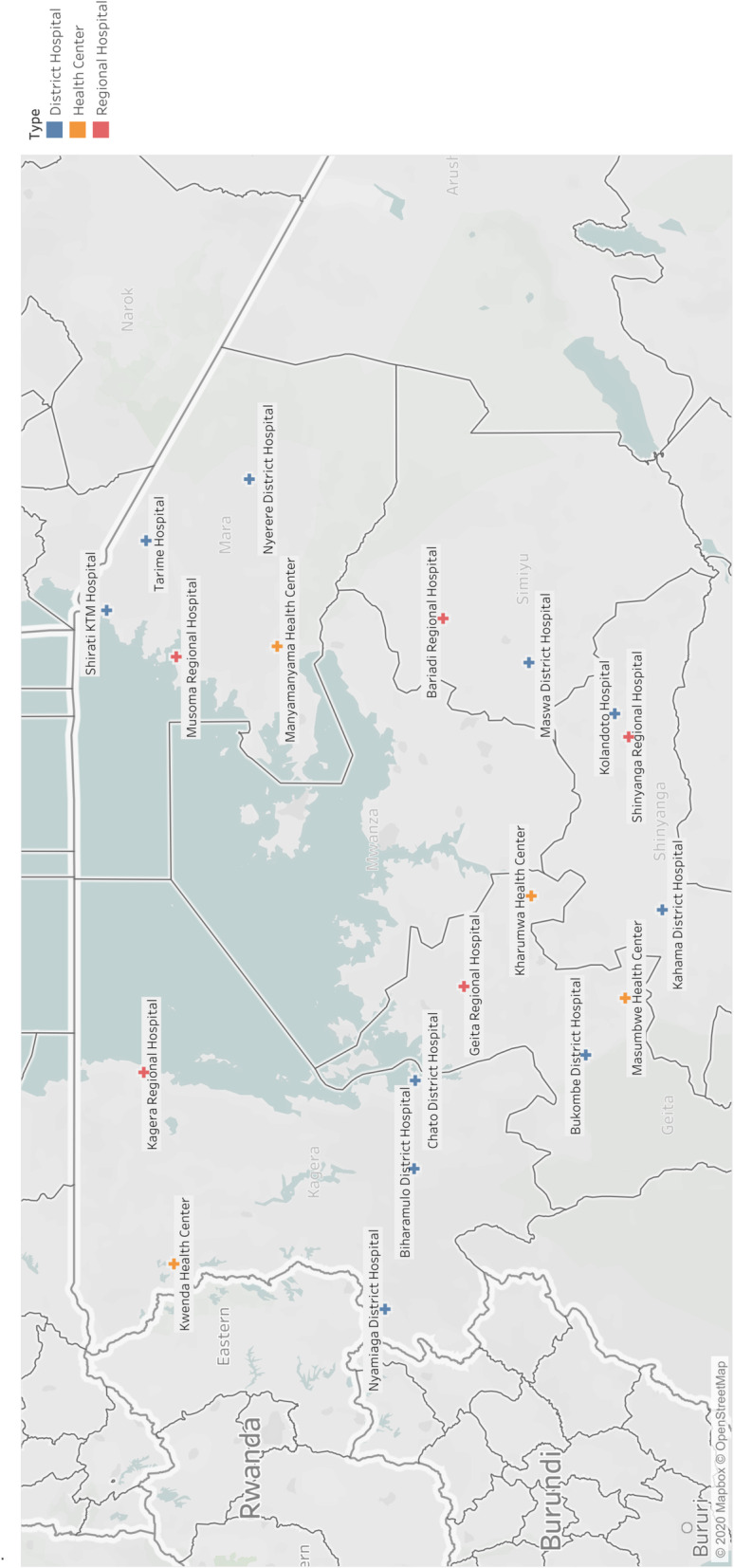
Distribution of sampled healthcare facilities. This figure was created by the authors using Google My Maps

The study was part of a larger prospective longitudinal quasi-experimental Safe Surgery 2020 (SS2020) study which aims to improve the quality of surgical care in Tanzania. Details on this study have been provided elsewhere [23]. Data collection was conducted from 1st February 2018 to 15th June 2018. A sample of 20 health facilities in 5 regions in the Lake Zone was chosen. This sample included four health centers, eleven district hospitals and five regional hospitals. For the purpose of the SS2020 study, Shinyanga and Simiyu were combined during sampling. Dispensaries were omitted from sample as they do not provide surgical services. Healthcare facilities were chosen based on the following criteria: 1) minimum average monthly surgical volume of 30 major surgical procedures 2) cross sectional representation of each level of the health system and 3) geographic distribution (Fig. 2). Information on services and basic infrastructure at all health facilities in the Lake zone was obtained from the MoHCDGEC’s health facility registry (HFR) and from the President’s Office for Regional and Local Government (PO-RALG) [22]. Based on this information, health facilities providing major surgical services in the five regions were selected. Geographic coordinates of health facilities from HFR were used to map health facilities using open source software QGIS (QGIS 2.4; QGIS Development Team; online resource).

### Data collection

As the study focused on surgical referrals, inpatient volume included all patients admitted into male, female and pediatric surgical wards as well as obstetric wards. Referral volume included all patients referred to any other health facility from these wards. Patients from non-surgical wards and those in the out-patient department were excluded from the study.

Details on referrals were captured by trained physician data collectors using a pre-tested data collection form (see Additional file 1). The data collected focused on patient demographics, reasons and destinations of referrals. Patient demographics captured included sex, age, type of management received and patient condition pre-referral (elective or emergency). More than one reason for referral could be selected per patient. For all referrals, data collectors also recorded details for why the specific reason for referral was selected as free-text. Furthermore, pre-referral diagnosis based on the best judgments of the referring healthcare provider and data collectors was noted for each patient referred.

Data on referrals were primarily collected from referral letters used by hospital staff for referrals. These referral letters typically contained information on pre-referral management, reason for referral and the facility to which the patient was being referred. This data was then extracted onto the standardized data collection form. To ensure the accuracy of the data captured in the health facility referral records, data collectors reviewed each referral and reason for referral with the clinical provider responsible for the referral on a daily basis. At health facilities where referrals letters were not routinely maintained, the standardized data set for the referral data collection form was obtained primarily by discussion with hospital staff. Data collectors did not provide any input on reason for referral or final decision to refer at any time. Rather, they aimed to capture the decisions made by the clinicians in the referral forms and through discussions with them.

Through discussions with clinicians, the data collectors also collected unstructured informal field notes on specific referrals to inform results collected. Field notes are not presented in the results section.

After completing daily data collection, all data was manually inputted into REDCap. Data quality checks were conducted by members of the study team through daily reviews on REDCap and weekly facility visits.

### Data analysis

Means and standard deviations were used to summarize numerical data while percentages and proportions were used to summarize categorical variables. Reasons for referrals were grouped into categories: staff, equipment, drugs and supplies, infrastructure and other reasons. Subgroup analysis by hospital level was performed on preventability, reasons and patterns for referrals.

The following formula was used to calculate referral rates:

$$ Referral\ rate=\frac{number\ of\ patients\ referred\ out\ from\ surgical\ and\ obstetric\ wards}{inpatients\ in\ surgical\ and\ obstetric\ wards} $$

In this study, an appropriate referral was defined as a referral to a higher level facility for a condition that should be treated at a facility higher than the referring facility as defined by the Tanzanian NSOAP. A preventable referral was defined as referral to a higher level facility for a condition that should be treated at the referring facility level defined using the same guidelines (see Fig. 1). To determine the preventability of each referral, three members of the research team with a clinical background assessed the recorded pre-referral diagnosis for each referral against the recommendations of the NSOAP for which surgical conditions should be treated at each hospital level. Conditions that were deemed non-surgical were not classified.

All analysis was performed in R Studio version 1.1.456 .

## Results

### Facility and patient characteristics

The inpatient volume in all surgical and obstetric wards over the study period was 35,317 with an average monthly inpatient volume per facility of 589. In total, 743 outward referrals were recorded giving an overall referral rate of 2.1%. The referral rate was highest at regional hospitals (2.9%), followed by district hospitals (1.9%) and health centers (1.5%).

Patient demographics are shown in Table 1. The most common pre-referral diagnoses were for suspected malignancies (18%) and fracture repairs (14%) (see Additional file 2).

**Table 1:** Characteristics of patients referrals from surgical and obstetric wards

Variables	Geita Region n (%)	Kagera Region n (%)	Mara Region n (%)	Shinyanga/Simiyu Regions n (%)	All regions n (%)
Age (mean, SD)	–	–	–	–	34.6 (24.2)
Average monthly inpatient volume	2623 (22.3)	3306 (28.1)	3297 (28.0)	2546 (21.6)	11,772 (100)
Sex					
Male	82 (51.2)	95 (66.0)	90 (66.2)	143 (47.2)	410 (55.2)
Female	78 (48.8)	49 (34.0)	46 (33.8)	160 (52.8)	333 (44.8)
Facility level					
Health Center	36 (22.5)	36 (25.0)	19 (14.0)	0 (0.0)	91 (12.2)
District Hospital	49 (30.6)	74 (51.4)	88 (64.7)	171 (56.4)	382 (51.4)
Regional Hospital	75 (46.9)	34 (23.6)	29 (21.3)	13 (43.6)	270 (36.3)
Pre-referral Management^a^					
Surgical	8 (4.9)	83 (55.0)	36 (24.8)	39 (12.8)	166 (22.3)
Medical	153 (93.3)	49 (34.0)	107 (73.8)	265 (86.6)	574 (77.3)
Obstetric	3 (1.8)	19 (12.6)	2 (1.4)	2 (0.7)	26 (3.5)
Pre-referral Condition					
Emergency	79 (49.4)	75 (52.1)	49 (36.0)	69 (22.7)	272 (36.6)
Elective	81 (50.6)	69 (47.9)	87 (64.0)	234 (77.2)	471 (63.4)
**Total**	160 (21.5)	144 (19.4)	136 (18.3)	303 (40.8)	743 (100)

^a^ Patients could receive more than one type of per-referral management

### Preventability of referrals

**Fig. 3:**
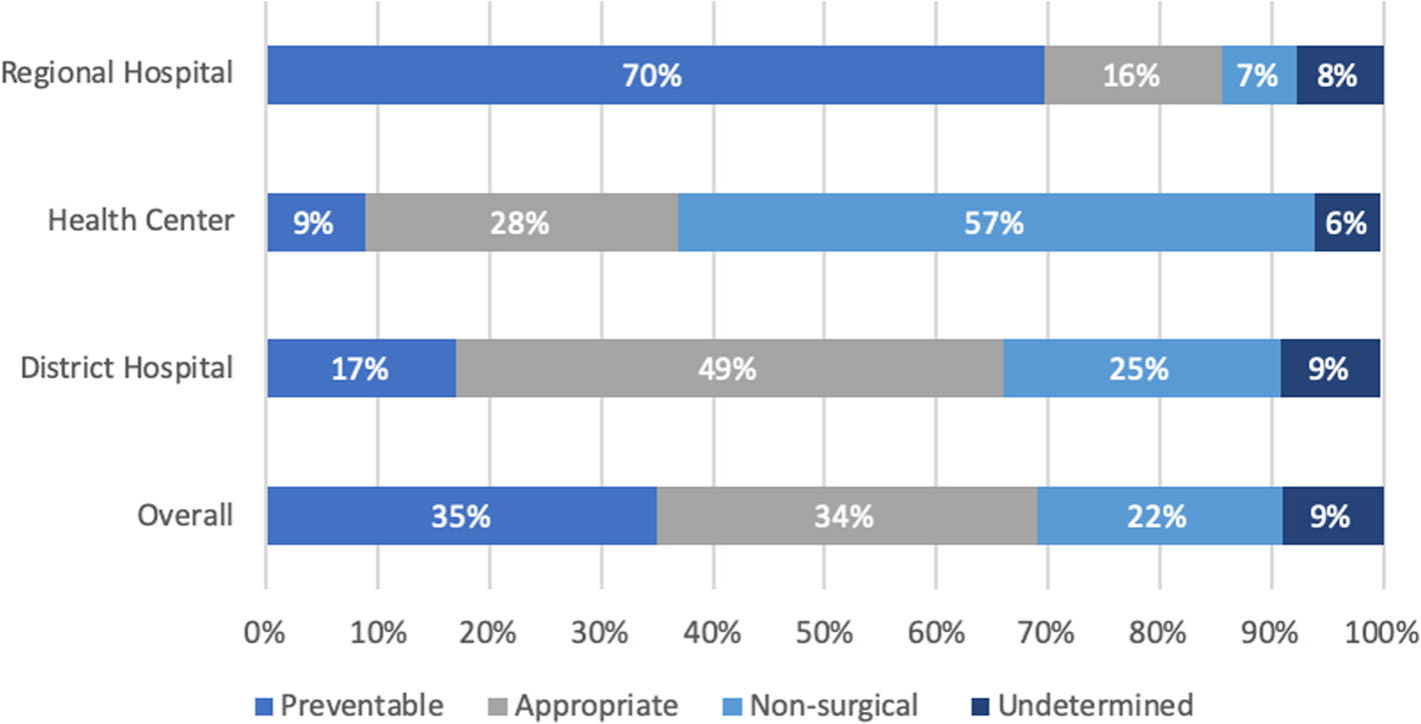
Appropriateness of referrals based on pre-referral diagnoses. This figure was created by the authors using Microsoft Excel for Mac version 16.29

Based on NSOAP recommendations of surgical procedures to be provided at each facility level and the recorded pre-referral diagnosis, 35% of all referrals were determined to be preventable while 34% were considered appropriate (Fig. 3). Twenty-two percent of all referrals were for non-surgical conditions. Health centers had the fewest preventable referrals (9%), followed by district hospitals (17%). A majority of the referrals from regional hospitals were considered preventable (70%).

### Reasons for referrals

The mean number of reasons for referrals per patient was 1.8 (SD 0.94). The reasons for referral are presented in Table 2. The most common reasons for referrals were staff-related (76.2%), followed by equipment (55.0%) and drugs or supplies (21.4%). Need for specialist care (72.9%), lack of imaging diagnostic equipment (38.1%) and lack of laboratory diagnostic equipment (24.9%) were common reasons for referral. A greater proportion of referrals at regional hospitals (16.3%) were due to lack of medical supplies and consumables than at district hospitals and health centers. More patients were referred for lack of drugs at district hospitals (11.5%) compared to health centers or regional hospitals while lack of blood was reported to be greatest at health centers (18.7%) compared to district and regional hospitals. Combined, less than 5% of all referrals were due to the lack of laboratory diagnostic training, patient preference, lack of utilities (water, electricity and oxygen), nonfunctional operating room, lack of inpatient beds and occupied operating room.

**Table 2:** Reasons for outward referral by initiating health facility level

Type of reason	Reason for outward referral	Initiating health facility level^a^			
		Health Centers *N* = 91 n (%)	District Hospitals *N* = 382 n (%)	Regional Hospitals *N* = 270 n (%)	Total *N* = 743 n (%)
Staff capacity	Need for specialist care	29 (31.9)	291 (76.2)	222 (82.2)	542 (72.9)
	Lack of post-operative care	0 (0.0)	18 (4.7)	16 (5.9)	34 (4.6)
	Need for imaging equipment training	7 (7.7)	6 (1.6)	6 (2.2)	19 (2.6)
	Member of surgical team not available	0 (0.0)	3 (0.8)	17 (6.3)	20 (2.7)
	Need for laboratory diagnostic training	0 (0.0)	6 (1.6)	2 (0.7)	8 (1.1)
Equipment	Lack of imaging diagnostic equipment	30 (33.0)	154 (40.3)	99 (36.7)	283 (38.1)
	Lack of laboratory diagnostic equipment	14 (15.4)	25 (6.5)	46 (17.0)	185 (24.9)
	Non-functional imaging diagnostic equipment	2 (2.2)	26 (6.8)	2 (0.7)	30 (4.0)
	Non-functional laboratory diagnostic equipment	0 (0.0)	15 (3.9)	0 (0.0)	15 (2.0)
Drugs and supplies	Lack of medical supplies and consumables	1 (1.1)	18 (4.7)	44 (16.3)	63 (8.5)
	Lack of drugs	7 (7.7)	44 (11.5)	5 (1.9)	56 (7.5)
	Lack of blood	17 (18.7)	19 (5.0)	4 (1.5)	40 (5.4)
	Lack of oxygen/water/electricity	1 (1.1)	5 (1.3)	0 (0.0)	6 (0.8)
Infrastructure	OR not functional	0 (0.0)	1 (0.3)	1 (0.4)	2 (0.3)
	Lack of inpatient beds	0 (0.0)	0 (0.0)	0 (0.0)	0 (0.0)
	OR occupied	0 (0.0)	0 (0.0)	0 (0.0)	0 (0.0)
Patient preference	Patient preference	2 (2.2)	6 (1.6)	2 (0.7)	10 (1.3)
Other	Other	0 (0.0)	1 (0.3)	0 (0.0)	1 (0.1)

^a^ Referrals total more than 100% because there could be more than one reason for referral per patient

Of the patients referred out for lack of specialist care, the most needed specialists reported were orthopedic surgeons (21%), urologists (12.5%) and neurosurgeons (7%) (Table 3).

**Table 3:** Referrals by specialty required

	Facility Level referred out from			
Specialist needed at referral	Health Center n (%)	District Hospital n (%)	Regional Hospital n (%)	Total n (%)
Orthopedic surgeon	3 (10.3)	39 (13.9)	69 (30.1)	111 (21.0)
Urologist	1 (3.4)	32 (4.6)	33 (14.4)	66 (12.5)
Neurosurgeon	0 (0.0)	13 (4.6)	25 (10.9)	38 (7.2)
Other specialist	8 (27.6)	22 (7.8)	3 (1.3)	33 (6.2)
Oncologist	2 (6.9)	12 (4.3)	15 (6.6)	29 (5.5)
Pediatrician	1 (3.4)	24 (8.5)	4 (1.7)	29 (5.5)
General surgeon	1 (3.4)	12 (4.3)	15 (6.6)	28 (5.3)
Obstetrician/Gynecologist	1 (3.4)	18 (6.4)	4 (1.7)	23 (4.3)
Cardiologist	1 (3.4)	16 (5.7)	4 (1.7)	21 (4.0)
Pediatric surgeon	0 (0.0)	8 (2.8)	13 (5.7)	21 (4.0)
Other surgical specialist	4 (13.8)	12 (4.3)	13 (5.7)	19 (3.6)
Otolaryngologist	1 (3.4)	9 (3.2)	7 (3.1)	17 (3.2)
Gastroenterologist	0 (0.0)	10 (3.6)	1 (0.4)	11 (2.1)
Physician	1 (3.4)	(1.4)	6 (2.6)	11 (2.1)
Anesthesiologist	0 (0.0)	7 (2.5)	2 (0.9)	9 (1.7)
Hematologist	1 (3.4)	5 (1.8)	3 (1.3)	9 (1.7)
Neurologist	2 (6.9)	6 (2.1)	1 (0.4)	9 (1.7)
Ophthalmologist	0 (0.0)	5 (1.8)	2 (0.9)	7 (1.3)
Intensivist	0 (0.0)	5 (1.8)	1 (0.4)	6 (1.1)
Dermatologist	0 (0.0)	2 (0.7)	3 (1.3)	5 (0.9)
Infectious Disease specialist	1 (3.4)	4 (1.4)	0 (0.0)	5 (0.9)
Cardiothoracic surgeon	0 (0.0)	1 (0.4)	2 (0.9)	3 (0.6)
Endocrinologist	1 (3.4)	2 (0.7)	0 (0.0)	3 (0.6)
Second surgeon opinion	0 (0.0)	3 (1.1)	0 (0.0)	3 (0.6)
Dental specialist (maxillofacial surgery)	0 (0.0)	1 (0.4)	2 (0.9)	3 (0.6)
Plastic surgeon	0 (0.0)	1 (0.4)	1 (0.4)	2 (0.4)
Psychiatrist	0 (0.0)	2 (0.7)	0 (0.0)	2 (0.4)
Thoracic surgeon	1 (0.0)	2 (0.7)	0 (0.0)	2 (0.4)
Missing data	2 (0.0)	1 (0.4)	0 (0.0)	1 (0.2)
Nephrologist	3 (0.0)	1 (0.4)	0 (0.0)	1 (0.2)
Physiotherapist	4 (0.0)	1 (0.4)	0 (0.0)	1 (0.2)
Pulmonologist	5 (0.0)	1 (0.4)	0 (0.0)	1 (0.2)
**Total**	**29 (100.0)**	**281 (100.0)**	**229 (100.0)**	**529 (100.0)**

Of the 261 surgical referrals identified to be preventable, the majority (78.2%) were due to the need for specialist care, followed by a lack of imaging diagnostic equipment (26.1%), lack of medical supplies and consumables (15.7%) and lack of laboratory diagnostic equipment (13.4%). Most of these preventable referrals were sent to the zonal hospital (70.0%).

### Referral patterns

Analysis of facilities to which patients were referred revealed that more than three quarters of all referrals (77%) were to the zonal hospital while 17% were to regional hospitals and 12% were to district hospitals. Table 4 summarizes the referral patterns and reasons for referral.

**Table 4:** Reasons for referral by receiving facility type

Type of reason	Reason for referral	Receiving health facility level^a^			
		Health Centers N = 2 n (%)	District Hospitals *N* = 89 n (%)	Regional Hospitals *N* = 116 n (%)	Zonal *N* = 518 n (%)
Staff capacity	Need for specialist care	0 (0.0)	21 (23.6)	77 (66.4)	436 (84.2)
	Lack of post-operative care	0 (0.0)	0 (0.0)	9 (7.8)	24 (4.6)
	Need for imaging equipment training	0 (0.0)	1 (1.1)	0 (0.0)	0 (0.0)
	Member of surgical team not available	2 (100.0)	0 (0.0)	3 (2.6)	15 (2.9)
	Need for laboratory diagnostic training	0 (0.0)	0 (0.0)	0 (0.0)	8 (1.5)
Equipment	Lack of imaging diagnostic equipment	0 (0.0)	21 (23.6)	30 (25.9)	228 (44.0)
	Lack of laboratory diagnostic equipment	0 (0.0)	7 (7.9)	28 (24.1)	143 (27.6)
	Non-functional imaging diagnostic equipment	0 (0.0)	25 (28.1)	2 (1.7)	3 (0.6)
	Non-functional laboratory diagnostic equipment	0 (0.0)	3 (3.4)	8 (6.9)	4 (0.8)
Drugs and supplies	Lack of medical supplies and consumables	0 (0.0)	2 (2.2)	8 (6.9)	51 (9.8)
	Lack of drugs	0 (0.0)	1 (1.1)	7 (6.0)	45 (8.7)
	Lack of blood	0 (0.0)	13 (14.6)	19 (16.4)	8 (1.5)
	Lack of oxygen/water/electricity	0 (0.0)	0 (0.0)	2 (1.7)	4 (0.8)
Infrastructure	OR not functional	1 (50.0)	1 (1.1)	0 (0.0)	0 (0.0)
	Lack of inpatient beds	0 (0.0)	0 (0.0)	0 (0.0)	0 (0.0)
	OR occupied	0 (0.0)	0 (0.0)	0 (0.0)	0 (0.0)
Patient preference	Patient preference	0 (0.0)	1 (1.1)	1 (0.9)	6 (1.2)
Other	Other	0 (0.0)	0 (0.0)	1 (0.9)	0 (0.0)

^a^ Referrals total more than 100% because there could be more than one reason for referral per patient

Two patients were referred downward from a regional hospital level to the health center level. The pre-referral diagnosis for both patients was obstetric complications. Analyses of receiving facility type and the reason for referral to that facility shows that the two patients referred to health centers were referred due to a member of the surgical team not being available. Thirty-six patients (9.4%) were referred horizontally to the same health facility level as their referring facility, the district hospital level.

## Discussion

This study examines surgical referrals in northern Tanzania in terms of preventability, reasons, rates and patterns in order to inform the implementation of the Tanzanian NSOAP and other health systems strengthening efforts. To our knowledge, this is the first study to document surgical referral rates in Tanzania.

We observed an average surgical referral rate of 2.1% through the study period, with referral rates highest at regional hospitals. It is difficult to determine whether these rates are high or low as changes in referral rates between facility and regions can be influenced by many factors, including population size, disease epidemiology, hospital capacity, population health-seeking behaviors and provider behaviors. Total referral rate benchmarks have been used in countries like Niger to monitor referral performance [24]. Ethiopia’s Saving Lives Through Safe Surgery policy recommends the tracking of referrals out [25]. Currently, Tanzania does not track referral rates. Tracking referral rates could be useful for monitoring the efficiency of the referral system, and to identify sources of inefficiencies and develop strategies to address them. However, referrals rates should be introduced with caution as such reporting may deter clinicians from making appropriate and necessary referrals in order to maintain low referral rates.

In this study, up to a third of all surgical referrals were found to be preventable, especially those from regional hospitals. Few studies document the preventability of referrals. In South Africa, Hollander and colleagues found that up to 40% of referrals from specialist hospitals were for conditions that could be managed at the referring hospital [7]. High rates of preventable or unnecessary referrals are problematic as they can overwhelm higher-level hospitals and reduce their capacity to meet the demand for more complex patient needs, for which they are designed. Understanding the reasons for preventable referrals is vital for designing strategies to reduce preventable referrals and improve the efficiency of the referral system.

Staff-related issues were the most common reasons for referrals in this study, with the majority of preventable referrals being due to the lack of specialists at the referring facility. This is perhaps understandable as a referral system is designed such that patients are referred up the referral chain for care which requires specialists, with specialized equipment and infrastructure. However, we find that most of the specialists for which most conditions are being referred are specialists that should be available at the referring facility type. For example, orthopedic surgeons were the most needed specialists accounting for a third of referrals from the regional hospitals and 14% of those referred from district hospitals. These findings align with findings by Simba and colleagues that reported a lack of expertise as a major reason for referral in Tanzania [13] and points to a deficiency in the current staffing capacity at the lower level facilities, particularly regional hospitals. This suggests that currently regional and district hospitals are not adequately staffed and/or skilled to provide the surgical services they are designed to provide. Other studies in Tanzania have equally noted a deficiency in surgical and anesthesia providers at lower levels [26–28]. Across the world staffing is often mentioned as a reason for preventable referrals [29, 30]. Upgrading regional hospitals to meet the Ministry of Health recommended staffing levels will likely reduce the number of unnecessary and preventable surgical and obstetric-related referrals.

Although more patients were referred for lack of drugs at the district hospitals compared to regional hospitals and health centers, overall, the percentage of referrals due to lack of drugs was low. This could either mean drugs, supplies, and infrastructure were readily available at the facility during the study period or that most patients were referred for other reasons, namely staff and diagnosing equipment, before drugs, supplies, and infrastructure were considered. The latter explanation is more plausible as previous studies have shown a severe shortage of drugs and supplies in Tanzania [13, 28, 31]. These have been shown to the most common reason for onward surgical referral in a recent systematic review of the reasons for surgical referral in LMICs [30]. Therefore, although these are not noted as major reasons for referrals in this study, the availability of drugs, supplies, and infrastructure should be taken into consideration when upgrading capacity at various levels of the referral system.

Patient preference was not a major reason for referral in this study. This may be due to patients bypassing the referral system to seek care directly at higher level facilities. Simba and colleagues found that at Muhimbili National Hospital in Dar es Salaam, up to 72.5% of admitted patients were self-referrals [13]. Patient self-referrals have been observed in Nigeria and South Africa where up to 92 and 50% of patients seen at secondary and tertiary level hospitals were self-referred [14, 15]. The higher rates in these studies compared with the 1.3% seen in this study relates to the fact that this study examines outward referrals from referring facilities, whilst other studies examine inbound referrals at receiving higher level facilities. Future studies should further explore patient-bypassing behaviors in Tanzania.

Although most patients were referred vertically up the referral pathway, some patients were referred horizontally to health facilities of the same level. Interestingly, field notes indicate that all such referrals were due to the lack of x-ray film at the initiating health facility. In Uganda, one study noted that patients were often referred due to the lack of drapes, surgical blades and gowns [5]. Although seemingly mundane, such issues can lead to significant delays in delivering care to the patient. This emphasizes the importance of maintaining functional diagnostic equipment at healthcare facilities.

**Fig. 4:**
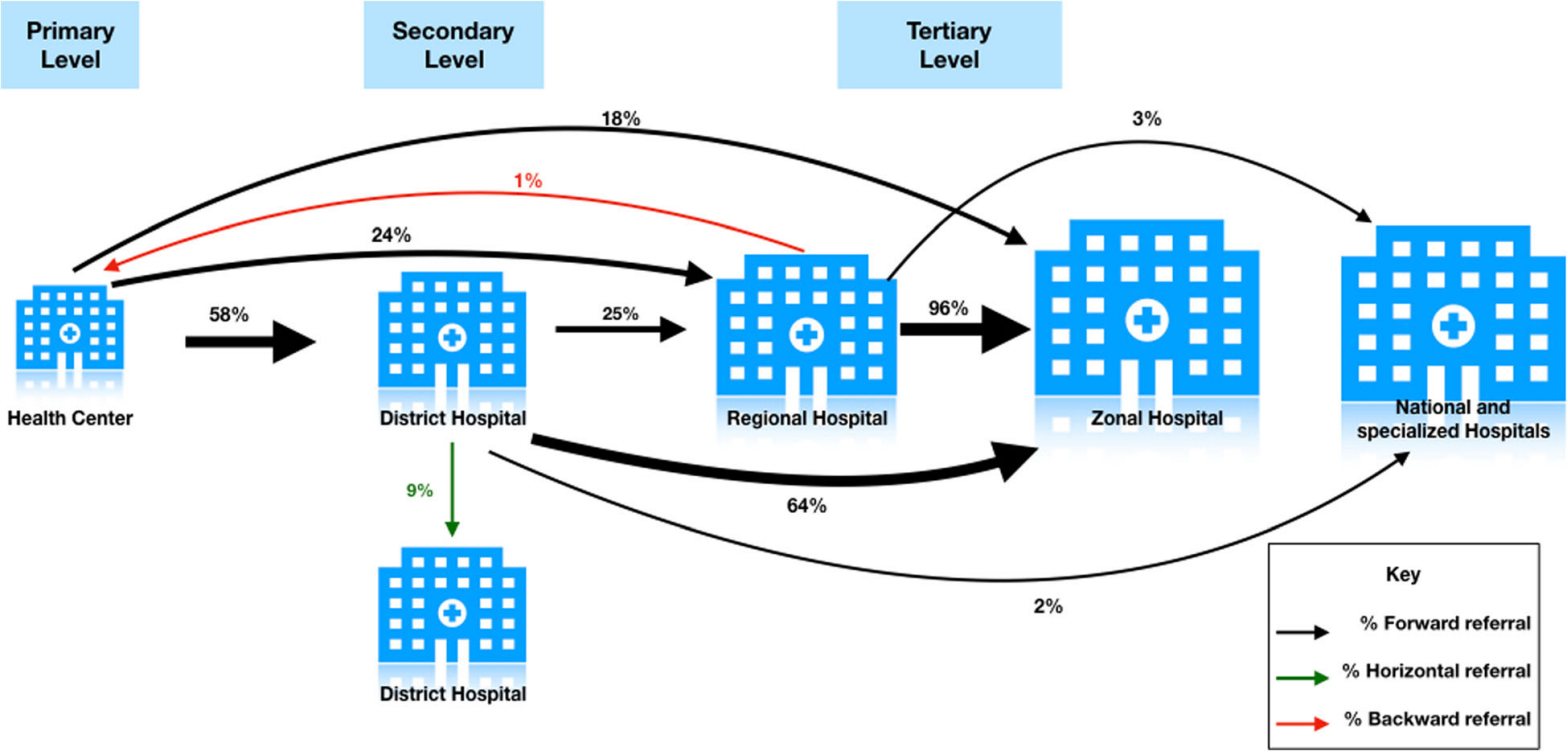
Patterns of referrals from surgical and obstetric wards. This figure was created by the authors using Keynote version 9.0.1 (6196)

As highlighted by Fig. 4, referral patterns in this study suggests that regional referral hospitals are significantly underutilized. Districts hospitals mostly refer to the zonal hospital bypassing the regional hospital while most referrals from regional hospitals, a majority of which are preventable, are referred to the zonal hospital, potentially overcrowding the zonal hospital. While emphasis has been placed on strengthening surgical capacity at district hospitals [32, 33], findings in this study suggests that rehabilitation of regional hospitals is also needed to provide surgical care closer to communities and reduce overcrowding at specialized hospitals.

Of note, there were two patients who were referred downward from a regional referral hospital to a health center. Both patients were urgently referred to a lower level facility as emergencies on two consecutive days because a member of the surgical team was absent. Field notes indicate that the missing member of the surgical team was an anesthetist who had fallen ill. There was no anesthetist to fill in for them resulting in a delay to patients and the eventual downward referral. This example highlights the acute shortage of anesthesia providers in Tanzania and a lack of flexibility in the system results in unpredictability of availability of services. Other studies have noted this as a major reason for self-referral to higher levels of care [5]. Currently, there are less than 50 physician anesthesia providers and about 180 non-physician anesthesia providers for a population of 53 million people equaling 0.09 anesthesia providers per 100,000 population [34]. This density falls significantly below the recommended 4–5 physician anesthesia providers per 100,000 population [34, 35]. Delays in surgical care provision resulting from a shortage of anesthesia care have been documented in Tanzania and other parts of sub-Saharan Africa [34, 36, 37]. Increasing the quantity and distribution of anesthesia providers is key to improving surgical capacity at lower level facilities [36–39].

Our study highlights the fact that improvements in the referral system will require a systemic approach. Healthcare facilities need to be sufficiently equipped and staffed to provide the surgical services required at their level. Though the most common reason for referral was staff-related, equipment-related reasons were also prominent. On average, about 2 reasons were given per patient referred. Therefore, focusing on increasing staff capacity at referral hospitals without improving diagnostic and laboratory capacity will likely not improve the efficiency of the referral system. Fifteen years on, our findings still resonate with findings by Simba and colleagues, which concluded that reducing overcrowding at tertiary level hospitals and improvement of the referral system are unlikely without improving service delivery capacity at primary and secondary care delivery levels.

### Limitations and strengths of the study

There are several limitations to this study that must be noted. It is possible that we underestimate the referral rates as some surgical and obstetric wards were not used solely for surgical and obstetric patients. For example, in some health facilities, medical patients were admitted into surgical and obstetric wards when all medical wards were at full capacity. As a result, the reported inpatient volume and referral rates may not represent surgical and obstetric patients exclusively. Thus, the referral rates must be interpreted in this light. Additionally, the most common site of referrals may be the emergency departments and outpatient units, as this is where new diagnoses are made and, therefore, where the decision whether or not the treatment can be undertaken is made. This is likely reflected in the fact that a majority of referrals were elective as the referrals for emergencies would not all have been captured. Patients referred for emergencies are those who may have the most time sensitive diagnoses and, therefore, experience the most harm from the delays of preventable referral. Nonetheless, the referral rates in this study provide plausible estimates for the proportion of patients referred from surgical and obstetric wards, as the majority of outward referrals warranted a surgical or obstetric intervention.

By design our study does not capture information on incoming referrals nor does it follow outward referrals to completion. Patients compliance to referral instructions is a crucial element of an efficient referral system, and one that is often lacking [18]. Examining referral compliance was, however, beyond the scope of our study. Future studies should examine compliance rates of surgical and obstetric patients in Tanzania.

It is also important to note that the assessment of preventability of surgical referrals were conducted using pre-referral diagnosis done at the referring facilities which may not always be accurate because of the poor diagnostic capabilities at lower level facilities. These diagnoses were provided based on the best clinical judgments of the referring clinicians and the medical data collectors.

Notwithstanding, this study provides useful information for improving Tanzania’s referral system. As far as we know, this is the only study conducted in Tanzania to date, that systematically examines the preventability, rates, patterns and reasons for referrals of surgical patients across different levels of the healthcare continuum. A major strength of this study is the rigor with which the information reported was collected. Many of the health facilities lack recordkeeping on referrals. Where available, these records are often inaccurate. To capture accurate data on referrals, trained medical data collectors were placed in study facilities. In addition to reviewing referral records at health facility, each referral was cross-checked by discussion with health staff to obtain details on referrals and discussions were exclusively used where referral records were not available. Daily online and weekly in-person data quality checks were performed by members of the study team. This allowed the trained data collectors to obtain the most accurate data on reasons and patterns of referrals.

## Conclusion

The most important finding from this study is that in order to improve the effectiveness of the health system in Tanzania through the referral system, overall human resource and infrastructure capacities need to be improved at lower level facilities, particularly regional referral hospitals where a majority of referrals are preventable. Though necessary, improving coordination and communication between health facilities alone will likely not be enough to strengthen the referral system. District and regional hospitals should be upgraded to meet minimum recommended staffing levels and be equipped to provide services required for their level of care as laid out in the Tanzania NSOAP.

## Supplementary information

**Additional file 1.** Referral-out data collection tool.

**Additional file 2.** Pre-referral diagnosis of patients in the study sites.

## Data Availability

The datasets and other material analyzed during the current study are available from the corresponding author on reasonable request.

## Abbreviations

HFR: Health Facility Registry
LMIC: Low-income and middle-income countries
MoHCDGEC: Ministry of Health, Community Development, Gender, Elderly and Children
NSOAP: National Surgical Obstetric and Anesthesia Plan
PoRALG: President’s Office, Regional Administration and Local Government
SS2020: Safe Surgery 2020

## References

[1] WHO | Health systems. WHO. [cited 2018 Oct 22]. Available from: http://www.who.int/topics/health_systems/en/.

[2] Kruk ME, Gage AD, Arsenault C, Jordan K, Leslie HH, Roder-DeWan S (2018). High-quality health systems in the sustainable development goals era: time for a revolution. Lancet Glob Health.

[3] WHO | Management of health facilities: Referral systems. WHO. [cited 2018 Oct 31]. Available from: http://www.who.int/management/facility/referral/en/.

[4] Haile T. Debas Peter Donkor Atul Gawande Dean T. Jamison Margaret E. Kruk Charles N. Mock. Disease Control Priorities, Third Edition (Volume 1): Essential Surgery. The World Bank; 2015. 442 p. (Disease Control Priorities). Available from: https://elibrary.worldbank.org/doi/abs/10.1596/978-1-4648-0346-8. 26740991

[5] Albutt K, Yorlets RR, Punchak M, Kayima P, Namanya DB, Anderson GA, et al. You pray to your God: A qualitative analysis of challenges in the provision of safe, timely, and affordable surgical care in Uganda. PLoS ONE. 2018 17;13(4). Available from: https://www.ncbi.nlm.nih.gov/pmc/articles/PMC5903624/. 10.1371/journal.pone.0195986 PMC5903624 29664956

[6] Goodman DM, Srofenyoh EK, Olufolabi AJ, Kim SM, Owen MD (2017). The third delay: understanding waiting time for obstetric referrals at a large regional hospital in Ghana. BMC Pregnancy Childbirth.

[7] den Hollander D, Albert M, Strand A, Hardcastle TC (2014). Epidemiology and referral patterns of burns admitted to the burns Centre at Inkosi Albert Luthuli Central Hospital. Durban Burns.

[8] Lee WS (2008). Pre-admission consultation and late referral in infants with neonatal cholestasis. J Paediatr Child Health.

[9] Nkurunziza T, Toma G, Odhiambo J, Maine R, Riviello R, Gupta N (2016). Referral patterns and predictors of referral delays for patients with traumatic injuries in rural Rwanda. Surgery..

[10] Meara JG, Leather AJM, Hagander L, Alkire BC, Alonso N, Ameh EA (2015). Global surgery 2030: evidence and solutions for achieving health, welfare, and economic development. Lancet.

[11] Cancer. World Health Organization. [cited 2018 Nov 12]. Available from: http://www.who.int/news-room/fact-sheets/detail/cancer.

[12] Road traffic injuries. World Health Organization. [cited 2018 Oct 31]. Available from: http://www.who.int/news-room/fact-sheets/detail/road-traffic-injuries.

[13] Simba DO, Mbembati NAA, Museru LM, Lema LEK (2008). Referral pattern of patients received at the national referral hospital: challenges in low income countries. East Afr J Public Health.

[14] Akande TM. Referral system in Nigeria: study of a tertiary health facility. Ann Afr Med. 2004;3(130-133).

[15] Mojaki ME, Basu D, Letskokgohka ME, Govender M (2011). Referral steps in district health system are side-stepped. SAMJ South Afr Med J.

[16] Font F, Quinto L, Masanja H, Nathan R, Ascaso C, Menendez C (2002). Paediatric referrals in rural Tanzania: the Kilombero District study – a case series. BMC Int Health Hum Rights.

[17] Ministry of health, Community Development, Gender, Elderly and Children. National Surgical, Obstetric and Anaesthesia Plan (NSOAP) 2018–2025. 2018 [cited 2018 Oct 31]. Available from: https://docs.wixstatic.com/ugd/d9a674_4daa353b73064f70ab6a53a96bb84ace.pdf.

[18] Pembe AB, Carlstedt A, Urassa DP, Lindmark G, Nyström L, Darj E (2010). Effectiveness of maternal referral system in a rural setting: a case study from Rufiji district, Tanzania. BMC Health Serv Res.

[19] World Bank. World Development Indicators | Data. [cited 2020 July 25]. Available from: https://data.worldbank.org/.

[20] Ministry of Health CD, MoH/Zanzibar M of H-, NBS/Tanzania NB of S-, OCGS/Zanzibar O of CGS-, ICF. Tanzania Demographic and Health Survey and Malaria Indicator Survey 2015-2016. 2016 [cited 2018 Oct 31]; Available from: https://dhsprogram.com/publications/publication-fr321-dhs-final-reports.cfm.

[21] Bugando Medical Centre. [cited 2018 Oct 31]. Available from: http://www.bugandomedicalcentre.go.tz/index.php?bmc=1.

[22] Ministry of health, Community Development, Gender, Elderly and Children. HFR WEB PORTAL - HomeAdvancedSearch Facilities. [cited 2018 Feb 10]. Available from: http://hfrportal.ehealth.go.tz/index.php?r=facilities/homeAdvancedSearch.

[23] Alidina S, Kuchukhidze S, Menon G, Citron I, Lama TN, Meara J, et al. Effectiveness of a multicomponent safe surgery intervention on improving surgical quality in Tanzania’s Lake Zone: protocol for a quasi-experimental study. BMJ Open. 2019 [cited 2019 Nov 10];9(10). Available from: https://bmjopen.bmj.com/content/9/10/e031800. 10.1136/bmjopen-2019-031800 PMC6797473 31594896

[24] Bossyns P, Abache R, Abdoulaye MS, Miyé H, Depoorter A-M, Van Lerberghe W (2006). Monitoring the referral system through benchmarking in rural Niger: an evaluation of the functional relation between health centres and the district hospital. BMC Health Serv Res.

[25] Federal Ministry of Health of Ethiopia. National Five Years Safe Surgery Strategic Plan. 2016. Available from: https://docs.wixstatic.com/ugd/d9a674_2ee52716f17f4ac4b1152f3b06aec61b.pdf.

[26] Cohen H, Cherian M, Groth S, Noel L, Mwakyusa DH, Penoyar T, et al. Emergency and surgery services of primary hospitals in the United Republic of Tanzania. BMJ Open. 2012;2(1). Available from: http://www.embase.com/search/results?subaction=viewrecord&from=export&id=L364379841 http://dx.doi.org/10.1136/bmjopen-2011-000369 http://sfx.hul.harvard.edu/sfx_local?sid=EMBASE&issn=20446055&id=doi:10.1136%2Fbmjopen-2011-000369&atitle=Emergency+and+surgery+services+of+primary+hospitals+in+the+United+Republic+of+Tanzania&stitle=BMJ+Open&title=BMJ+Open&volume=2&issue=1&spage=&epage=&aulast=Penoyar&aufirst=Tom&auinit=T.&aufull=Penoyar+T.&coden=&isbn=&pages=-&date=2012&auinit1=T&auinitm=. 10.1136/bmjopen-2011-000369 PMC3274714 22307096

[27] Pereira C, Mbaruku G, Bergström S, McCord C, Nzabuhakwa C (2011). Emergency obstetric surgery by non-physician clinicians in Tanzania. Int J Gynecol Obstet.

[28] Ministry of Health and Social Welfare. Tanzania Service Availability and Readiness Assessment (SARA) 2012. 2013 [cited 2017 Dec 29]. Available from: https://ihi.eprints.org/2448/1/SARA_2012_Report.pdf.

[29] S C, H P, Aa H, F A, E S, H O, et al. Assessment of Capacity for Surgery, Obstetrics and Anaesthesia in 17 Ghanaian Hospitals Using a WHO Assessment Tool. Tropical medicine & international health : TM & IH. 2010 [cited 2019 Nov 28]. Available from: https://pubmed.ncbi.nlm.nih.gov/20636302/. 10.1111/j.1365-3156.2010.02589.x 20636302

[30] C P, R B, J G. Surgical Referral Systems in Low- And Middle-Income Countries: A Review of the Evidence. PloS one. 2019 [cited 2019 Nov 28]. Available from: https://pubmed.ncbi.nlm.nih.gov/31560716/?from_term=Surgical+referral+systems+in+low-+and+middleincome+countries%3A+A+review+of+the+evidence&from_pos=1. 10.1371/journal.pone.0223328 PMC6764741 31560716

[31] Nyberger K, Jumbam DT, Dahm J, Maongezi S, Makuwani A, Kapologwe NA, et al. The Situation of Safe Surgery and Anaesthesia in Tanzania: A Systematic Review. World J Surg. 2018; Available from: 10.1007/s00268-018-4767-7. 10.1007/s00268-018-4767-7 PMC6313359 30128771

[32] al GM et. Essential surgery at the district hospital: a retrospective descriptive analysis in three African countries. - PubMed - NCBI. [cited 2018 Jan 14]. Available from: https://www-ncbi-nlm-nih-gov.ezp-prod1.hul.harvard.edu/pubmed/?term=Essential+surgery+at+the+district+hospital%3A+A+retrospective+descriptive+analysis+in+three+african+countries. 10.1371/journal.pmed.1000243 PMC2834708 20231871

[33] E M, C H, Bl H-G, J O, R M, N G, et al. Non-Obstetric Surgical Care at Three Rural District Hospitals in Rwanda: More Human Capacity and Surgical Equipment May Increase Operative Care. World journal of surgery. 2016 [cited 2019 Dec 2]. Available from: https://pubmed.ncbi.nlm.nih.gov/27098541/?from_term=surgical+care+at+district+level&from_pos=1. 10.1007/s00268-016-3515-0 PMC4982876 27098541

[34] Kempthorne P, Morriss WW, Mellin-Olsen J, Gore-Booth J (2017). The WFSA global anesthesia workforce survey. Anesth Analg.

[35] Davies JI, Vreede E, Onajin-Obembe B, Morriss WW (2018). What is the minimum number of specialist anaesthetists needed in low-income and middle-income countries?. BMJ Glob Health.

[36] Ashengo T, Skeels A, Hurwitz EJH, Thuo E, Sanghvi H. Bridging the human resource gap in surgical and anesthesia care in low-resource countries: a review of the task sharing literature. Hum Resour Health. 2017;15. Available from: https://www.ncbi.nlm.nih.gov/pmc/articles/PMC5688799/. 10.1186/s12960-017-0248-6 PMC5688799 29115962

[37] Dubowitz G, Twagirumugabe T, Lugazia E, Chokwe TM, Epiu I, Mijumbi C (2017). Challenges of anesthesia in low- and middle-income countries: a cross-sectional survey of access to safe obstetric anesthesia in East Africa. Anesth Analg.

[38] Epiu I, Tindimwebwa JVB, Mijumbi C, Chokwe TM, Lugazia E, Ndarugirire F (2017). Challenges of anesthesia in low- and middle-income countries: a cross-sectional survey of access to safe obstetric anesthesia in East Africa. Anesth Analg.

[39] Larsson E, Eriksson J, Baker T (2014). Quality of anaesthesia for caesarean sections at Muhimbili National Hospital, Dar Es Salaam, Tanzania. Eur J Anaesthesiol.

