# Supplementary figures and images for: Surgical referrals in Northern Tanzania: a prospective assessment of rates, preventability, reasons and patterns
Source: BMC Health Serv Res. 2020 Aug 8;20:725. doi: 10.1186/s12913-020-05559-x (PMC7414731; doi:10.1186/s12913-020-05559-x)

# Additional File 1

**Referral-out data collection tool**


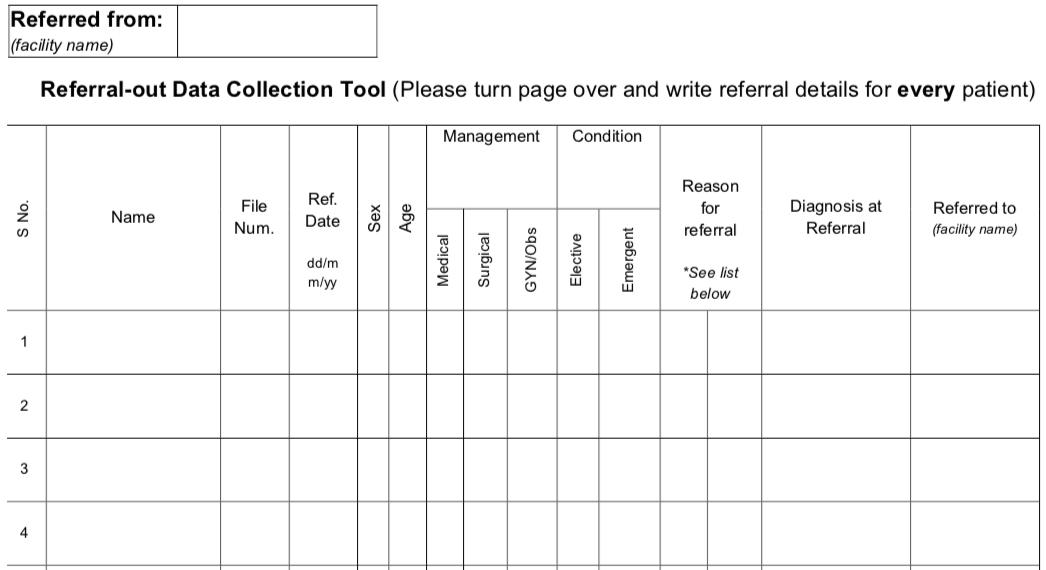


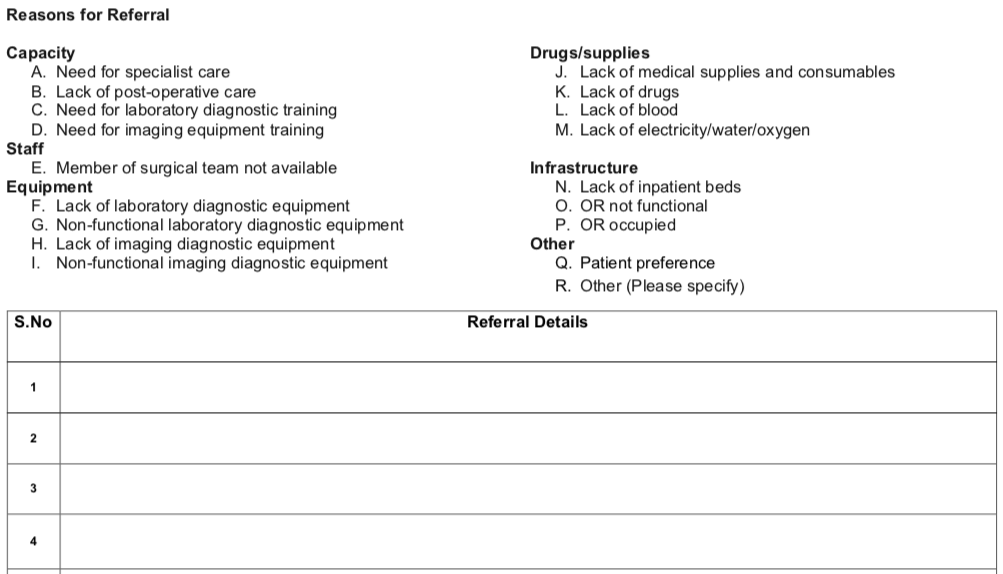

Supplement: Supplementary file 1 — Additional file 1. Referral-out data collection tool. [file 12913_2020_5559_MOESM1_ESM.docx]
