# Supplementary material for: Surgical referrals in Northern Tanzania: a prospective assessment of rates, preventability, reasons and patterns
Source: BMC Health Serv Res. 2020 Aug 8;20:725. doi: 10.1186/s12913-020-05559-x (PMC7414731; doi:10.1186/s12913-020-05559-x)
Supplement: Supplementary file 2 — Additional file 2. Pre-referral diagnosis of patients in the study sites. [file 12913_2020_5559_MOESM2_ESM.docx]

**Additional file 2: Pre-referral diagnosis of patients in the study sites**

| **Diagnosis** | **Number of referrals** | **Percentage of total referrals (%)** |
| --- | --- | --- |
| Suspected malignancies | 136 | 18% |
| Fractures | 106 | 14% |
| Anemia | 47 | 6% |
| Brain/ Head Injury | 47 | 6% |
| Gastrointestinal disorders | 32 | 4% |
| Intestinal Obstruction | 32 | 4% |
| Nephrological / Urological disorders | 31 | 4% |
| Heart failure | 26 | 3% |
| Infectious diseases | 26 | 3% |
| Obstetric complications | 24 | 3% |
| Spine and spinal cord injuries | 21 | 3% |
| Stroke | 19 | 3% |
| Other trauma and injuries | 15 | 2% |
| Other congenital disorders | 14 | 2% |
| ENT disorders | 13 | 2% |
| Osteomelytis | 12 | 2% |
| Thoracic disorders | 12 | 2% |
| Other | 11 | 1% |
| Gynecological disorders | 10 | 1% |
| BOO | 9 | 1% |
| Other Haematological diseases | 9 | 1% |
| Diabetes | 8 | 1% |
| Other orthopedic conditions | 8 | 1% |
| Peritonitis | 8 | 1% |
| Hypertensive diseases | 7 | 1% |
| Malnutrition | 7 | 1% |
| Neurological disorders | 7 | 1% |
| Other heart/ vascular system diseases | 7 | 1% |
| Congenital heart disease | 6 | 1% |
| Foreign body removal | 6 | 1% |
| Appendicitis | 4 | 1% |
| Neonatal conditions | 4 | 1% |
| Opthalomological disorders | 4 | 1% |
| Psychiatric diseases | 4 | 1% |
| Extremity wounds/ulcers | 3 | 0% |
| Dermatological disorders | 2 | 0% |
| Missing | 1 | 0% |
| Acute abdomen | 1 | 0% |
| Hepatocellular | 1 | 0% |
| LUMBER SACRAL SPONDYLOSIS | 1 | 0% |
| Metabolic disorders | 1 | 0% |
| Renal stones | 1 | 0% |
